# Dynamics of Classical Swine Fever Spread in Wild Boar in 2018–2019, Japan

**DOI:** 10.3390/pathogens9020119

**Published:** 2020-02-13

**Authors:** Norikazu Isoda, Kairi Baba, Satoshi Ito, Mitsugi Ito, Yoshihiro Sakoda, Kohei Makita

**Affiliations:** 1 Unit of Risk Analysis and Management, Research Center for Zoonosis Control, Hokkaido University, Kita 20, Nishi 10, Kita-Ku, Sapporo 001-0020, Japan; isoda@czc.hokudai.ac.jp (N.I.); satoshi125@czc.hokudai.ac.jp (S.I.); 2 Global Station for Zoonosis Control, Global Institute for Collaborative Research and Education (GI-CoRE), Hokkaido University, Sapporo 001-0020, Japan; sakoda@vetmed.hokudai.ac.jp; 3 Veterinary Epidemiology Unit, School of Veterinary Medicine, Rakuno Gakuen University, 582, Bunkyodai Midorimachi, Ebetsu 069-8501, Japan; s21561088@stu.rakuno.ac.jp; 4 Akabane Animal Clinic, Co. Ltd., 55 Ishizoe, Akabane-Cho, Tahara 441-3502, Japan; m-ito@oasis.ocn.ne.jp; 5 Laboratory of Microbiology, Department of Disease Control, Faculty of Veterinary Medicine, Hokkaido University, Kita 18, Nishi 9, Kita-Ku, Sapporo 060-0818, Japan

**Keywords:** classical swine fever, Japan, space–time analysis, wild boar

## Abstract

The prolongation of the classic swine fever (CSF) outbreak in Japan in 2018 was highly associated with the persistence and widespread of the CSF virus (CSFV) in the wild boar population. To investigate the dynamics of the CSF outbreak in wild boar, spatiotemporal analyses were performed. The positive rate of CSFV in wild boar fluctuated dramatically from March to June 2019, but finally stabilized at approximately 10%. The Euclidean distance from the initial CSF notified farm to the farthest infected wild boar of the day constantly increased over time since the initial outbreak except in the cases reported from Gunma and Saitama prefectures. The two-month-period prevalence, estimated using integrated nested Laplace approximation, reached >80% in half of the infected areas in March–April 2019. The area affected continued to expand despite the period prevalence decreasing up to October 2019. A large difference in the shapes of standard deviational ellipses and in the location of their centroids when including or excluding cases in Gunma and Saitama prefectures indicates that infections there were unlikely to have been caused simply by wild boar activities, and anthropogenic factors were likely involved. The emergence of concurrent space–time clusters in these areas after July 2019 indicated that CSF outbreaks were scattered by this point in time. The results of this epidemiological analysis help explain the dynamics of the spread of CSF and will aid in the implementation of control measures, including bait vaccination.

## 1. Introduction

Classical swine fever (CSF) is a highly contagious disease causing a multisystemic infection in domestic and wild pigs. CSF is distributed worldwide and causes enormous economic losses in husbandry due to its high virulence in domestic pigs [1]. The causative agent of CSF is the CSF virus (CSFV), which belongs to the genus *Pestivirus* and the family *Flaviviridae*. CSFV exhibits a variety of disease modes in host animals with infections that may be acute, subacute, chronic, late-onset, or asymptomatic. It is known that disease severity depends on the virulence of the CSFV, age and species of a host animal, and status of individual or herd immunity. CSFVs with moderate virulence have recently been isolated in Mongolia and China [2,3].

Japan once achieved the elimination of CSF through the application of the attenuated CSFV vaccine [4]. Since 1992, no notifications of CSF had been reported, and Japan was designated as a CSF-free country by the World Organisation for Animal Health (OIE) in 2007 [4]. However, in September 2018, CSF reemerged in Gifu Prefecture, and despite strenuous control efforts, the outbreak was not successfully contained. Detection and culling and movement restriction, which are all basic control measures for CSF outbreaks in domestic pigs, were implemented. However, due to the wider spread of the disease, the government decided to apply preventive vaccination in domestic pigs in the affected prefectures in October 2019 to inhibit further CSF spread. The current CSF outbreaks were indicated to be driven by the circulation of a CSFV with a moderate pathogenicity that most closely matched in identity in two regions of CSFVs recently isolated in China and Mongolia, thereby further complicating the outbreak situation [4,5]. A high proportion of dead wild boars found in the affected areas were positive for CSFV infection, even in the early phase of the current CSF outbreak [6]. For this reason, prefectural offices in and around the affected area decided to implement an intensive program to capture wild boar for CSFV testing and to erect fencing to control wild boar movements. Moreover, due to the further spread of CSF from the prefectures affected, the Japanese government decided to apply oral bait vaccination in selected areas of the affected prefectures in three seasons of 2019. The initial batch of bait was disseminated twice between March and May 2019 in two prefectures (Aichi and Gifu). The second batch of bait was disseminated twice (in most prefectures) between July and September 2019 in nine prefectures (Gifu, Aichi, Mie, Fukui, Nagano, Toyama, Ishikawa, Shizuoka, and Shiga). Despite the control measures targeting wild boar, the trend of CSF infection was not terminated. As of the end of November 2019, there were 50 CSF outbreaks in pig farms, leading to the death of approximately 120,000 animals in seven prefectures, along with 1470 cases in wild boar in 12 prefectures [6,7].

One year after the initial CSF notification, the lack of success in controlling the outbreak is concerning. To provide another perspective that could be of assistance, we investigated the dynamics of CSF spread in wild boar by analyzing the transmission pattern of CSFV in wild boar temporally and spatially. The identification of CSF cases which were unlikely to have been transmitted via wild boar would suggest important opportunities for biosecurity measures in farms and a disease containment strategy.

## 2. Results

### 2.1. Temporal Trend of CSF Cases in Wild Boar

**Figure 1:**
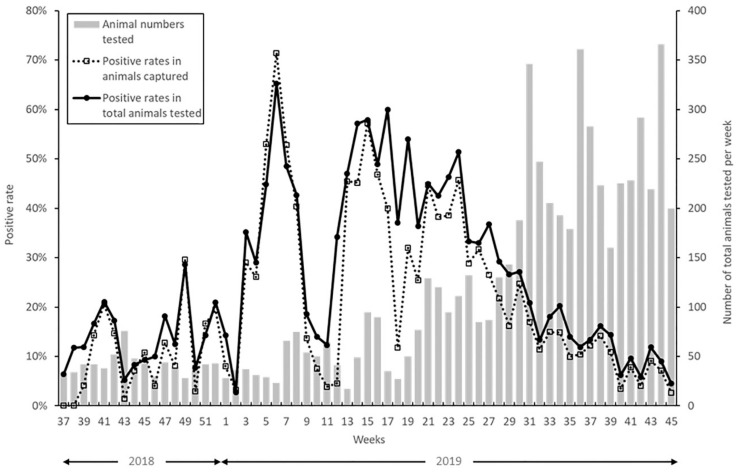
Positive rates of classical swine fever virus (CSFV) in wild boar. Results for CSFV antigen detection in wild boar in 12 prefectures were combined. Solid line: CSFV-positive rates in total animals tested in each week. Dashed line: CSFV-positive rates in animals captured in each week. Bar chart: The number of dead and captured wild boars tested in each week.

From September 2018 to the middle of November 2019, a total of 6,594 wild boars, including 826 dead and 5768 captured animals were tested for CSF infection (Figure 1). After the utilization of bait vaccination, no wild boars positive for a CSFV strain used for the oral vaccine were reported. During the early phase of the CSF outbreak, from the initial notification to the end of 2018, in which cases were limited in two prefectures, the positive rates ranged mostly between 10% and 20%. The fluctuation was larger in the first half of 2019 and, especially between March and June 2019, the ratio increased to between 40% and 60%. However, as the number of tested animals increased, the CSFV-positive rates decreased gradually to approximately 10% in the second half of 2019.

### 2.2. Distance of CSF Cases in Wild Boar from the Initial Outbreak Point

**Figure 2:**
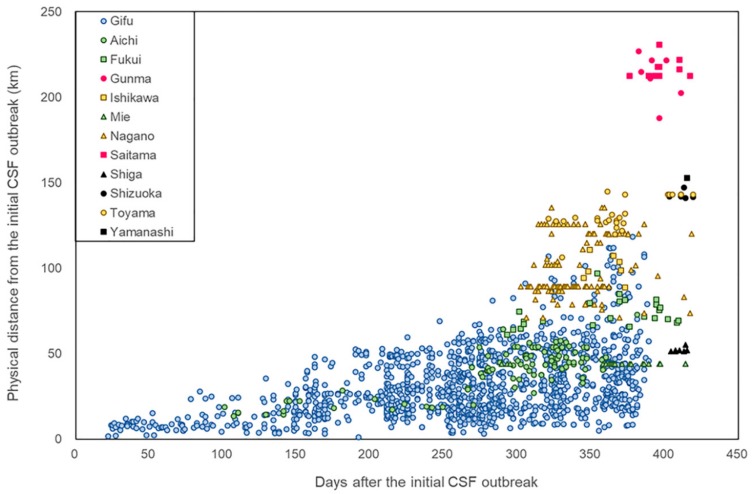
Distances of CSF cases in wild boar from the initial case over time.

In general, the direct distance between the locations of the initial CSF notification and cases in wild boar increased proportionally with time (Figure 2). However, many of the notifications reported in the second half of 2019 did not correspond with the general trend, with those from Saitama and Gunma prefectures in particular appearing unexpectedly distant from the initial location in a rather short time.

For each case of CSF notified in wild boar, the distance from the initial CSF case and time since the initial case was plotted.

### 2.3. Spatial Change of CSF Period Prevalence Over Time

**Figure 3:**
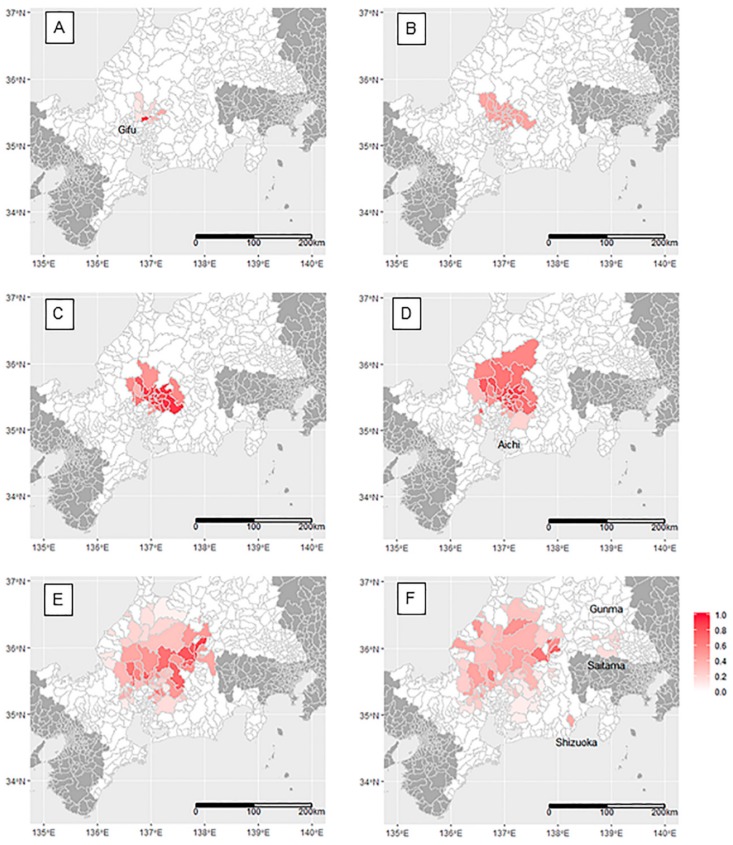
Spatial change of two-month-period prevalence of CSF in wild boar.

The two-month-period prevalence showed the first peak around the area of the initial farm case in November–December 2018 (Figure 3A). The mean period prevalence in 10 infected municipalities was 32.5% (median = 21.2%, 95% credible interval: CI of median posterior = 9.0%–42.0%), and distinct high period-prevalence was observed in two municipalities: 90.0% (95% CI: 68.0%–99.7%) and 81.6% (95% CI: 40.7%–99.7%). The expanded infected areas (19 municipalities) had moderate homogeneous prevalence, with mean, median, and interquartile ranges of period-prevalence estimates at 44.0%, 45.0%, and 44.9%–45.1%, respectively, in January–February 2019 (Figure 3B). The prevalence reached over 80% in half of the infected areas (11 of 22 municipalities) in March–April 2019 (Figure 3C). The mean, median, and interquartile ranges of the estimates were 73.7%, 79.3%, and 59.0%–88.4%, and the 95% CI of median posterior was 52.7%–95.5%. As the infected areas continued to expand, the period prevalence began to reduce until the end of the period of observation in September–October 2019 (72 municipalities maximum, Figure 3D–F). The mean, median, and interquartile ranges of the period prevalence estimates in September–October 2019 were 27.4%, 24.8%, and 15.7%–35.4%. In this period, the disease in wild boar was detected in remote municipalities (not contiguous with the existing infected area) in Saitama and Gunma, as well as in Shizuoka prefecture (Figure 3F).

The intensity of the red color indicates the estimated two-month-prevalence of CSF in wild boar at the municipality level, with an intensity of 1.0 indicating a prevalence of 100%. A: November–December 2018, B: January–February 2019, C: March–April 2019, D: May–June 2019, E: July–August 2019, F: September–October 2019.

### 2.4. Standard Deviational Ellipse Analysis

**Figure 4:**
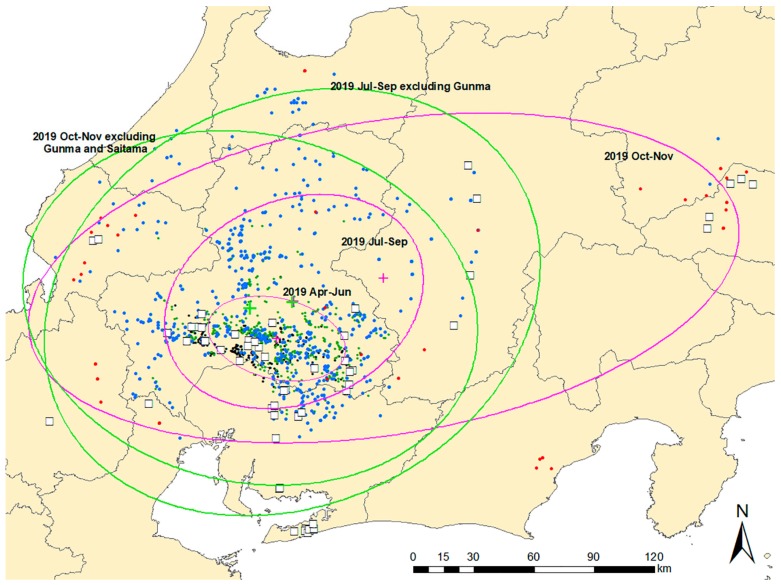
Spatiotemporal distribution of classical swine fever notifications from April to November 2019.

Standard deviational ellipses (SDEs) for the three phases were overlaid on a map with CSF-positive notifications to illustrate the directional trends and dispersion of CSF notifications (Figure 4). The position of the centroids of the ellipses in the two early phases did not differ on the map, whereas the centroid of the third ellipse was positioned approximately 100 km away from the other two and toward the northeast. The forms of the ellipses for the second and third periods, excluding disease notifications in Gunma and Saitama prefectures, differed from those that included these notifications. The centroids of the ellipses for the second and third periods, excluding the notifications in the two eastern provinces, were located relatively close to the centroid for the first period.

Standard deviational ellipses for three time periods (April–June 2019, July–September 2019, October–November 2019). Ellipses and their centroids (green and pink plus signs) were overlaid with CSF notifications distinguishing domestic pig (white square) and wild boar (dot) cases. Black dot: CSF notification of wild boar in September 2018–March 2019, green dot: April–June 2019, blue dot: July–September 2019, red dot: October–November 2019. For the last two periods, standard deviational ellipses that exclude the notification data in Gunma and Saitama prefectures are shown.

### 2.5. Space–Time Cluster Analysis

**Figure 5:**
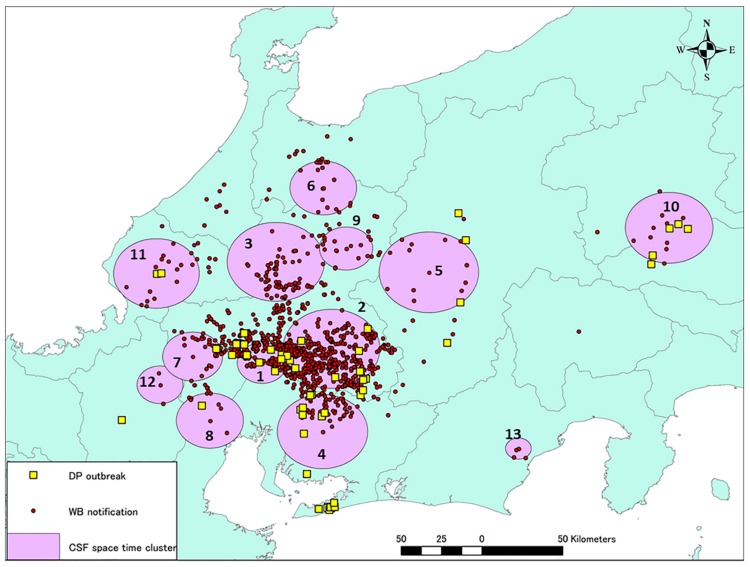
Locations of the significant space–time clusters of CSF.

A total of 13 significant space–time clusters were identified from the CSF notification dataset by space–time permutation analysis based on the 26-km upper limit on cluster size set in the software (Figure 5). Clusters 1 and 2 equate to the periods September 2018 to February 2019 and February to June 2019, respectively, and their timings did not overlap (Table 1). However, after June 2019, several clusters appeared concurrently in different areas with the disease scattered widely, including in the two eastern provinces. Compared to Cluster 1, the radii of the clusters identified after June 2019 were greater but the durations were shorter, indicating that the disease was being disseminated rapidly and widely even within the cluster areas. The habitats of each of the 13 clusters were visually assessed with the guide of Global Map Specifications [8]. Most of the areas in the clusters comprised several types of forests and croplands. 

During the study period, from September 2018 to November 2019, 13 significant clusters were observed in or around the disease notification area. Detailed information for each cluster is given in Table 1. Yellow squares indicate the locations of CSF-positive farms. Red circles indicate the locations of CSFV-positive wild boar either captured or found dead.

## 3. Discussion

At the time of writing, November 2019, more than one year has passed since the initial CSF notification in September 2018, and the outbreak has still not been terminated. In this period, 46 CSF outbreaks in pig farms were reported with approximately 120,000 killed animals, but the wild boar population was considered to play a critical role in the spread of the disease. Disease notifications were concentrated at locations near the initial cases in the early phase of the current outbreaks and then became more widespread over time. The results from the present study indicate that the disease could have spread via the movement of wild boar to nearby contiguous areas was confirmed from spring 2019 onward through spatial changes in period prevalence. The risk of CSFV infection at a farm located at a 5-km distance from a CSFV-positive wild boar within 28 days was estimated at more than 5% in Hayama et al. [9]. It is noteworthy that the disease became dispersed to remote municipalities in Gunma and Saitama prefectures (areas that were not contiguous with the main outbreak), which was unexpected given the occurrence and spread patterns of CSFV in wild boar. In the SDE analysis, this unexpected dispersion of CSFV to two distant prefectures was demonstrated by a shift in the centroids and shape distortion of the ellipses between October and November 2019. In the Gunma and Saitama area, the first CSFV infection was confirmed in a pig farm before the detection of CSF cases in wild boar. Given the epidemiological situation, as well as the results of the epidemiological analysis in the present study, it seems likely that CSF jumped to Gunma and Saitama prefectures by factors other than transmission by wild boar without being detected. The phylogenetic analysis also supports that the CSFVs isolated in the current outbreak indicated that the CSFV isolated in the first farm in Saitama prefecture was most close to the strain isolated in Aichi prefecture, which was adjacent to neither the Gunma nor Saitama prefecture [6]. At this time, no epidemiological relevance between the CSF positive farms in these two prefectures and ones in other prefectures, including the introduction of potentially infected pigs, have been revealed. The spontaneous introduction of infectious pathogens by the movement of fomites, including humans and vehicles from the high-risk areas, might be a possible pathway of the CSF jump. Though the details will be revealed in the further epidemiological investigation, these would be associated with poor biosecurity measures in farms to introduce the contagious pathogens, or with low compliance in wild boar trapping to acquire the pathogens. Poor biosecurity measures in farms, including imperfect change of clothes and shoes and incomplete disinfection, as well as imperfect installation of fencing with large mesh that allow small animals passing, could also contribute to the introduction of the pathogen agent inside the farm. Furthermore, when wild boars are trapped for sample collection for laboratory diagnosis, adequate hygienic sampling and animal transportation, as well as intensive disinfection of clothes, equipment, and environment around the captured animal, are critical to minimize the level of contamination of the environment in order to prevent secondary infections in wild boar during capturing activities. Biosecurity measures in farms and wildlife management activities against CSFV should be reviewed to prevent careless facilitation of transmissions in both and between domestic pig and wild boar populations.

Since the 1990s, wild boar has been recognized as an important reservoir of CSFV due to a change in pathogenicity from high to moderate virulence in wild boar as well as domestic pigs. Transmission routes of CSFV are comparable in wild boar and domestic pigs, and occur either through direct contact between diseased animals or indirectly via feces, food, and carcasses [10]. During the 1993–1998 CSF outbreak in Germany, an indirect transmission of CSFV to domestic pigs from wild boar was indicated [11]. The infection of wild boar with moderately virulent CSFV enables a more effective transmission to other animals, and once a CSFV with moderate virulence crosses into the wild boar population, the disease becomes prevalent and persistent among unmonitored populations. It was also reported that CSFV tended to persist and become endemic for years in larger wildlife populations [12]. As population size and density are considered crucial factors for CSFV survival in wild boar populations [13], much effort has been focused toward population management, including hunting and trapping. However, it has also been demonstrated that a depopulation strategy is not effective for CSF control in wildlife because of the low probability of achieving depopulation to the desired low level, high uncertainty in the estimation of the number of wild boar, and low acceptability for depopulation among hunters [14]. Furthermore, hunting has been reported to play a negative role in CSF control in wild boar because excessive hunting pressure might increase population turnover, enabling the maintenance of pathogens among younger naïve animals and causing population mixing, leading to more frequent contact among animals. Delivery of bait vaccines has been considered effective as a control measure to limit CSF spread in wildlife by decreasing the proportion of susceptible animals. Although prophylactic vaccination is banned in Europe, the application of preventive vaccination is allowed in domestic pigs and wild boar if the spread of disease appears to be uncontrollable [10]. Bait vaccines for wild boar were employed during CSF outbreaks in Germany and France [15,16]. The estimations of the ideal vaccination rate in wild boar for the control of CSF were reported as 41% using a deterministic model, or from 9% to 52% using a stochastic model based on an outbreak of CSF in Pakistan [17,18].

Bait vaccination with the commercial vaccine (Pestiporc, Oral, IDT Biologika GmbH, Dessau-Rosslau, Germany) for wild boar was utilized twice between March and May 2019 in selected areas in Aichi and Gifu prefectures where CSF positive cases were found [19]. Oral vaccination of wild boar is an effective tool to decrease the number of susceptible animals against CSFV in the affected area with relatively low costs. Oral mass vaccination of wild boar against CSF has been conducted since the late 1990s in some European countries [14]. Thirty or forty baits each were delivered at 660 (in March) and 1,011 (April to May) locations, respectively, in these two prefectures. The overall bait collection rate after five days was 41.4%, and wild boar bite-mark traces were observed in approximately 25% of the remaining collected baits. On this basis, it was estimated that the intake rate of bait vaccine in the wild boar population was, at maximum, approximately 70%. The positive rate for CSFV antibodies increased from 50% before baiting to 70% after baiting within the vaccinated area in Aichi prefecture and from 40% to 62% in Gifu prefecture. However, care needs to be taken for a comparative interpretation of the effectiveness of the vaccination in two prefectures due to differences in the diagnosis and sampling methods (personal communication). The results of the spatial change of CSF period prevalence in wild boar in May–June 2019 when there was an expansion in the area of CSFV-positive wild boar demonstrated that the oral vaccination program was not able to prevent the spread of CSF, but worked on reducing prevalence in heavily affected areas. According to the results of the present study, CSFV might have been circulating at the early phase of the outbreak (from the initial case to April 2019) among wild boar in a limited area (Figure 3). From January to April 2019, the disease did not spread to a wider area, but was transmitted to more sensitive animals inside the existing area, resulting in CSFV infection of over 80% of the wild boar. Because the movement of wild boar is restricted mainly by snowfall in winter, and most Japanese wild boar (*Sus scrofa leucomystax*) breed piglet in April-June, especially in May, it would have been necessary to complete bait vaccination in the affected areas no later than May 2019 when the CSFV spread further by the movement of the wild boar [20,21,22]. In addition, comprehensive guideline for the vaccination of wild boar against CSFV at a national level, describing the methods of sample size calculation, sampling, and diagnosis for the evaluation, should be established. Since decreasing the sensitive wild boar to prevalent CSFV is so critical to achieving the containment of a CSF outbreak in domestic pigs, the development of an effective vaccination strategy for wildlife with practical and effective guidelines and adequate implementation should be highly prioritized. The sampling and diagnostic strategies for CSFV detection in wild boar are currently varied among prefectures. The lack of unified comprehensive guideline might also influence the interpretation of the results of CSFV detection from wild boar, like the cases in the Shizuoka prefecture. Though in Figure 5, all the CSF cases in wild boar in Shizuoka prefectures were geographically isolated and clustered, it seems likely that the CSF was transmitted to the Shizuoka prefecture by wild boar. This is because, in Figure 1, the CSF cases in wild boar in Shizuoka prefecture mostly corresponded with the associations between the direct distance from the location of the initial CSF notification and cases in wild boar. Sampling and diagnostic bias would conceal the dynamics of disease spread by expressing “non-positive” results.

## 4. Conclusions

CSFV infection in domestic pigs was continuously notified in Japan since September 2018 and spread more widely mainly through wild boar movement. The implementation of effective control measures in wildlife, such as bait vaccination under a well-planned strategy and the involvement of a surveillance program using hunting or a capture scheme, is essential for successful containment. Though biosecurity measures were strengthened at pig farms to prevent CSF introduction, unexpected outbreaks occurred in pig farms in areas where the wild boar were unlikely to have been infected with CSFV. The current control measures both for domestic pigs and wild boar should be intensively reviewed.

## 5. Materials and Methods

### 5.1. Data and Data Sources

Epidemiological data of CSF notification and reverse transcription polymerase chain reaction (RT-PCR) test results of CSFV detection in domestic pigs and wild boar between September 9, 2018, and November 15, 2019, were collected from the websites of 15 prefectures. In Japan, the RT-PCR based on the Vilcek et al., using a positive control of the attenuated CSFV strain GPE^−^, is performed in Livestock Hygiene Service Centers under the direction of the National Institute of Animal Hygiene as one of the diagnostics of CSFV detection [23,24]. The coordinates (latitude and longitude) of the CSF notifications were obtained from the website of the OIE [7]. A total of 1418 CSF notifications, 48 outbreaks on domestic pig farms, and 1370 cases in wild boar were confirmed during this period [7], as well as 5324 wild boars that were negative for CSFV infection. As we focused on the local spread of CSFV, notifications of CSF by slaughtering or in facilities through which CSF-affected pigs had been transported were not included in the present study.

### 5.2. Temporal Trend and Linear Distance of CSF Cases in Wild Boar from the Initial Case

The dates and locations of CSFV detection from both dead-found and captured wild boars were used to investigate the relationship between the time elapsed and distance from the location of the initial CSF notification in the domestic pig farm to each of the CSF cases in wild boar. The dates and locations of wild boars tested for CSFV, including those produced negative results, were used for the calculation of weekly positive rates of CSFV among both dead-found and captured animals, and among only captured animals, respectively, to describe the temporal trend of CSF positive rates in wild boar in expanding infected areas.

### 5.3. Description of Spatial Change of CSF Prevalence Over Time

Two-month-period wild boar diagnostic positive and negative results based on PCR tests were aggregated at the municipality level for the period between September 2018 and October 2019, and the period prevalence in each administrative unit was estimated using an integrated nested Laplace approximation (INLA) with zero-inflated binomial errors using the package R-INLA in the statistics software R version 3.6.1 (R Core Team, 2019) [25]. Intrinsic conditional autoregression (CAR) was selected to deal with spatial autocorrelation, based on the lowest value of deviance information criteria among the latent models in R-INLA.

### 5.4. SDE Analysis

SDE analysis was performed to describe the trend and spatial characteristics of CSF notifications in the study area using ArcGIS v10.6.1 software (ESRI Inc., Redlands, CA, USA). This provided the orientation and shape of a distribution, and dispersion of the diseases in domestic pigs and wild boar, following an approach similar to those in previous studies [5,26,27]. The ratio of the long and short ellipse axes was used to identify the degree of clustering or dispersion. To analyze the temporal changes in CSF notifications since July 2019, the study period was divided into three phases: (i) April to June 2019, (ii) July to September 2019, and (iii) October to November 2019.

### 5.5. Multi-Distance Spatial Cluster Analysis and Space–Time Cluster Analysis

A multi-distance spatial cluster analysis tool in ArcGIS v10.6.1 was used to identify the maximum distance of the relationships between CSF notifications by applying the common transformation of Ripley’s *K* function. Detailed information on the method for calculating the maximum distance of relationships, which yielded the highest Diff *K* value, was described in a previous study [5]. A space–time permutation technique was applied to examine the presence of space–time clusters in the area affected by CSF. The upper limit on the geographical size of the cluster was set to 26 km, the minimum time aggregation to seven days, and the maximum temporal cluster size to 50% of the total study period (default setting) [28]. A Monte Carlo process was implemented using 999 replications to test for the presence of candidate clusters (*p* < 0.05). Analyses were conducted in SaTScan software v9.6 (Kulldorff, Boston, MA, USA) [29]. The habitat of each cluster was visually assessed with the guide of Global Map Specifications to assess the pattern of land cover in the cluster identified [8].

## Figures and Tables

**Table 1:** Details of each space-time cluster detected (*p* < 0.05) in CSF notification.

Cluster	Duration (Days)	Start Date	End Date	Radius (km)	Main Land Covers *
1	148	2018/9/9	2019/2/4	12.53	Closed shrublands, Cropland/Natural vegetation mosaic
2	132	2019/2/12	2019/6/24	24.41	Mixed forest
3	69	2019/6/25	2019/9/2	24.33	Mixed forest, Deciduous broadleaf forest, Cropland/Natural vegetation mosaic
4	48	2019/7/2	2019/8/19	22.88	Mixed forest, Deciduous broadleaf forest
5	48	2019/7/16	2019/9/2	24.89	Cropland/Natural vegetation mosaic, Mixed forest
6	13	2019/9/3	2019/9/16	16.59	Deciduous broadleaf forest
7	27	2019/9/3	2019/9/30	15.11	Mixed forest, Deciduous broadleaf forest
8	55	2019/9/3	2019/10/28	16.99	Closed shrublands, Mixed forest, Croplands
9	20	2019/9/3	2019/9/23	13.30	Cropland/Natural vegetation mosaic, Mixed forest
10	41	2019/10/1	2019/11/11	21.87	Closed shrublands, Croplands, Mixed forest
11	27	2019/10/1	2019/10/28	21.46	Croplands, Deciduous broadleaf forest
12	20	2019/10/15	2019/11/4	11.61	Water bodies, Croplands, Mixed forest
13	20	2019/10/15	2019/11/4	6.52	Mixed forest

*: Types of main land covers in the cluster were visually assessed with Global Map Version 1.2.1 Specifications [8] and sorted in descending order of types.
